# Blunt-chest-trauma-induced acute myocardial infarction

**DOI:** 10.4322/acr.2021.263

**Published:** 2021-05-25

**Authors:** Senthil Kumar, Yogender Singh Bansal, Nikhil Mehta, Ritambhra Nada, Pulkit Girdhar, Vikarn Vishwajeet

**Affiliations:** 1 Postgraduate Institute of Medical Education and Research, Department of Forensic Medicine, Chandigarh, India; 2 Postgraduate Institute of Medical Education and Research, Department of Histopathology, Chandigarh, India; 3 All India Institute of Medical Sciences, Department of Pathology and Laboratory Medicine, Jodhpur, India

**Keywords:** Thoracic Injuries, Atherosclerotic Plaque, Coronary Thrombosis, Myocardial Infarction, Morphological and Microscopic Findings

## Abstract

Blunt chest trauma (BCT) is one of the rarest causes of acute myocardial infarction (AMI). This paper reports the case of a young married man who suffered from AMI due to BCT sustained in a fight with his wife. The histopathology examination revealed a rupture of atherosclerotic plaque with superimposed thrombus in the proximal left anterior descending artery. This report also reviews previously reported BCT-induced AMI cases in the literature.

## INTRODUCTION

Acute myocardial infarction (AMI) is usually a complication of coronary artery disease (CAD). The well-known risk factors are hypertension, diabetes mellitus, smoking, obesity, and poor diet habits.^1^ Blunt chest trauma (BCT) is known to cause cardiac lesions, such as pericardial injury, cardiac contusion, avulsion of cardiac valves, cardiac ruptures, and cardiac tamponade. However, BCT causing AMI is rare: only a handful of cases are reported in the literature.^2^Although rare, BCT is one of the non-atherosclerotic mechanisms that lead to acute myocardial infarction in young patients, often without a clinical history of heart disease.

## CASE REPORT

**Figure 1:**
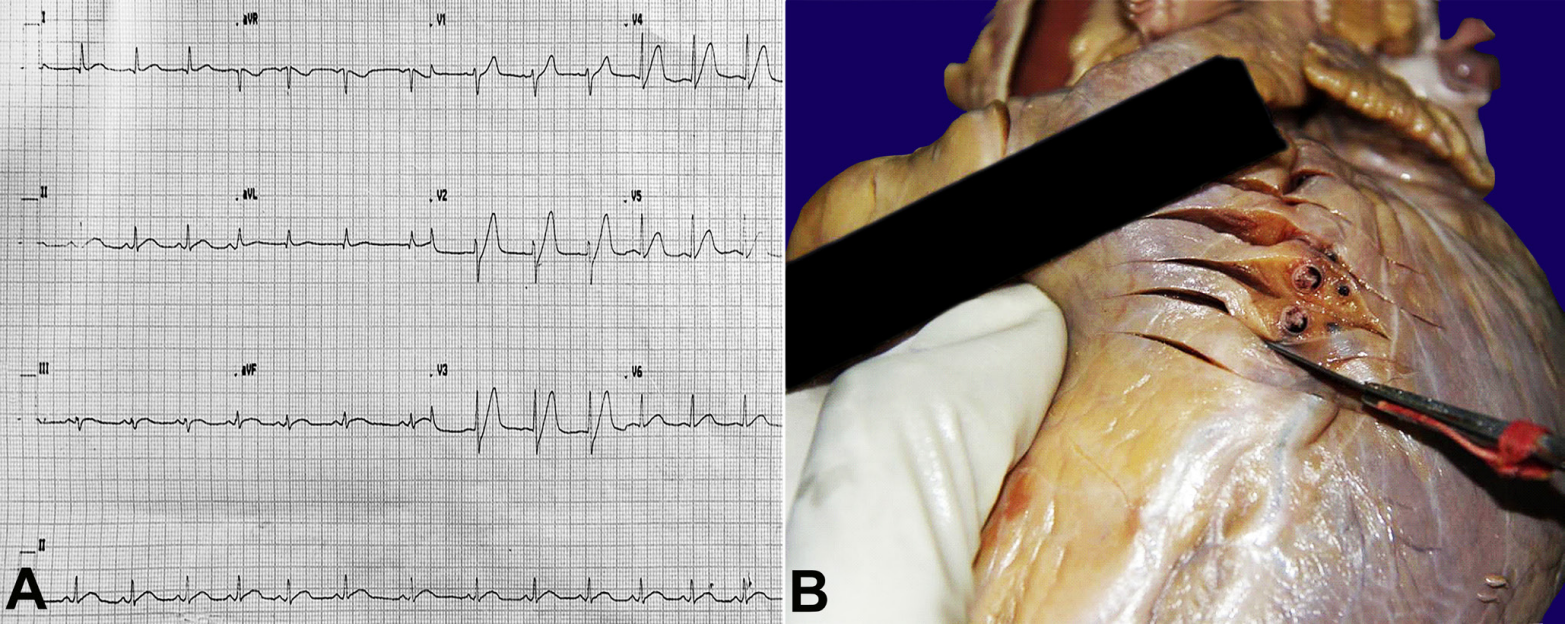
ECG and gross findings. **A –** ECG showing hyperacute T waves in V1, V2, and V3 leads; **B –** Occlusion of LAD artery at its proximal part. ECG = electrocardiogram; LAD = left anterior descending

A 32-year-old male with a history of assault was brought to the emergency department by his neighbor. He was allegedly hit with a mop handle by his wife during a quarrel and sustained chest and head trauma in that scuffle. On admission, he complained of diffuse chest pain radiating to the left shoulder. On examination, his Glasgow Coma Scale (GCS) score was 15, blood pressure 110/70 mmHg, pulse rate 88 per minute, and respiratory rate 20 per minute. His chest X-ray and computed tomography (CT) showed no abnormality. Cardiology consultation was taken, and the electrocardiogram (ECG), performed 3 hours after admission, showed sinus rhythm with a ventricular rate of 84 beats per minute. The cardiologist observed early repolarization changes in ECG (Figure 1A) and the troponin T level was within normal limits. With initial inconclusive evidence of acute coronary syndrome (ACS), the patient was evaluated for head injury. 8 hours after admission, the patient suddenly collapsed, and his vitals were not recordable. The patient was immediately intubated; cardiopulmonary resuscitation (CPR) was started and continued for 45 minutes. Despite the resuscitative efforts, the patient died and was declared dead at 9:15 AM. The autopsy was performed because of the legal implications of the case.

## AUTOPSY FINDINGS

On autopsy, multiple curvilinear to linear reddish abrasions suggestive of nail marks were found on the face, neck, both forearms, and hands. Also, a reddish contusion of 2cm × 0.5cm and an abrasion of 2cm × 0.8cm were found on the front of the left side of the chest, just below the level of the nipple, near the sternum. On internal examination, the fourth and fifth ribs were found fractured along the midaxillary line on the left side. In the absence of rib fractures in antemortem chest x-ray and CT, the possibility of fracture due to CPR was considered. Approximately 60 mL of blood-tinged fluid was found in the left pleural cavity. There were no contusions on either lung. On opening the pericardium, there was no blood in pericardial cavity; the heart weighed around 400 g (reference range [RR];230-270 g) and showed discoloration of the left ventricular wall. The root of the aorta showed multiple atherosclerotic patches. On examination of the coronaries, the proximal part of the left anterior descending (LAD) artery showed obstruction. The whole heart was preserved in 10% neutral buffered formalin for further gross and histopathological examination. On opening the skull cap, there was no intracranial hemorrhage, and the brain did not show any hemorrhage or contusion. The abdominal organs were grossly normal.

## GROSS AND HISTOPATHOLOGICAL EXAMINATION

Gross examination of the whole heart was done in the presence of trained pathologists, by inflow and outflow technique, and extensive sampling was performed from all the cardiac chambers. All coronaries were dissected at 1 cm serial cuts and sampled for histopathology. On gross examination of the formalin-fixed heart, the right atrium and ventricle appeared unremarkable. The left ventricular wall, interventricular septum, and apex showed a diffuse area of discoloration consistent with an extensive myocardial infarction (MI). A thrombus was identified in the proximal part of the LAD occluding 80% of the lumen (Figure 1B). However, the left main coronary, left circumflex, and right coronary artery appeared normal. There were multiple atherosclerotic patches over the arch of the aorta and its branches. The mitral, aortic, pulmonary and tricuspid valves were unremarkable.

**Figure 2:**
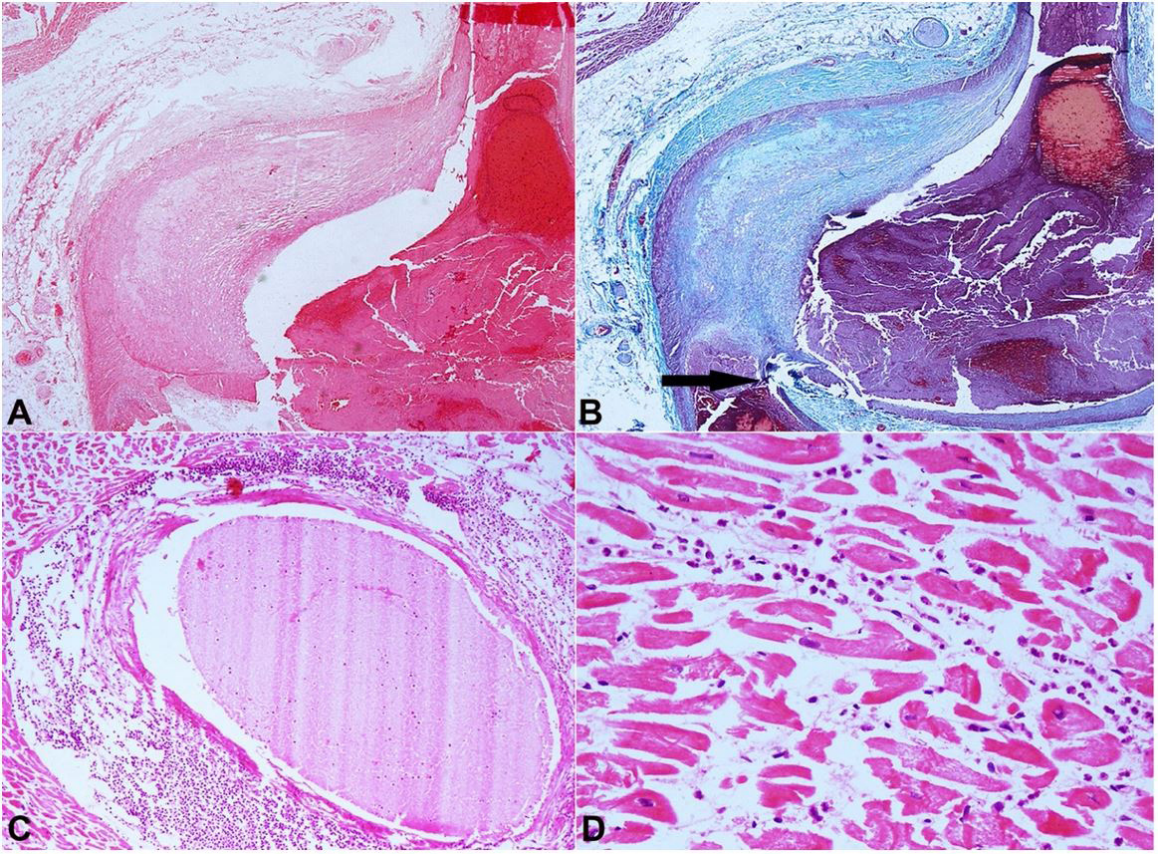
Photomicrographs of the heart. **A** – Low-power magnification shows marked intimal thickening of the left anterior descending artery wall and fresh fibrin thrombi in the arterial lumen (H&E, 20X); **B –** Masson trichrome stain highlights the markedly thickened intima as light green color (20X). The arrow points to the intimal plaque rupture with hemorrhage; **C –** The myocardium shows perivascular collections of neutrophils which are also spilling into surrounding the cardiomyocytes (H&E, 40X); **D –** Scattered neutrophils within the myocardium causing destruction of the myocytes. Also note the loss of nuclei and presence of contraction bands in these fibers (H&E, 100X).

On microscopic examination, sections showed large ruptured intimal plaque in the left anterior descending artery with fresh fibrin thrombi in its lumen. Fibrin thrombus was confirmed with phosphotungstic acid and martial scarlet blue stain. The lumen appeared to be occluded by the thrombus, which was also propagating along the lumen. The left ventricular wall showed occasional foci of perivascular neutrophilic collections around medium-sized vessels. In addition, loss of cardiomyocytes with edema, contraction bands, and early infiltration of neutrophils were noted (Figure 2). The gross and microscopic findings suggested that the MI occurred 4 to 24 hours before death. We performed the Elastic von Gieson stain, and no pre-existing vascular anomalies/vasculitis were identified. The toxicology test results were negative for alcohol, drugs, and other common poisons. The concluding opinion was that early MI caused death due to occlusion of the left anterior descending artery by a ruptured atherosclerotic plaque with superimposed thrombus as a consequence of blunt trauma to the chest.

## DISCUSSION

The deceased was a 32-year-old married well-built male with no previous history of cardiac illness, hypertension, or diabetes mellitus. He was a smoker and a social drinker. On the day of the incident, a minor fight erupted between himself and his wife. During this quarrel, he was attacked with force by his wife using a mop handle, and he sustained a blunt trauma to his chest. On autopsy, the blunt trauma was evident in the form of abrasions and contusions on the left side of his chest. The conclusion was that blunt force trauma to the chest likely caused the rupture of the pre-existing atherosclerotic plaque in the LAD artery, which, in turn, led to thrombus formation. The ruptured intimal plaque together with the superimposed thrombus obstructed the lumen and led to acute MI in this case. It is interesting to note that the LAD artery is the most frequently injured coronary artery in blunt chest trauma due to its anatomical location. The LAD artery runs in the anterior surface of the heart; thus, this artery is vulnerable to external impact.^2^

In fact, on admission, the deceased complained of left-side chest pain radiating to the left shoulder, which is typical of angina. Based on this complaint, he was evaluated with ECG and cardiac troponin (cTn). The ECG was interpreted as early repolarization changes, and the troponin T level taken 3 hours after the alleged incident was negative. With these initial inconclusive screening results for acute coronary syndrome, and this being a case of trauma, the patient was evaluated along the lines of head injury. Further, the follow-up with repeat ECG and troponin levels were missed in this case. When we reviewed the deceased’s medical records after the autopsy, the ECG report findings were cross-checked. We consulted the physician to review the ECG findings, and he reported hyperacute T waves in the V1,V2, and V3 leads (Figure 1A) suggestive of evolving anterior wall MI. A hyperacute T wave is one of the earliest ECG signs of acute ischemia, which subsequently evolves as ST-segment elevation.^3^ It may be seen as early as 30 minutes after the chest pain.^4^ Since these ECG findings can also be seen in hyperkalemia, left ventricular hypertrophy, and an early repolarization variant, it is essential to rule out every possibility to diagnose it as AMI.^5^ Once the patient presents with a hyperacute T wave with chest pain typical of angina, symptomatic treatment with nitroglycerin or morphine and oral antiplatelet agents should be started. The administration of unfractionated heparin should be considered, and the patient should be continuously monitored by frequent serial 12-lead ECG and cTn.^6^ In our case; ideally, a serial ECG should have been performed, and a cTn test should have been repeated 6 hours after the admission to rule out myocardial ischemia.^7^ ^,^ ^8^ In cases where the MI closely follows the BCT, the angina may be misinterpreted as pain due to thoracic injury sustained in the trauma. This misinterpretation with early inconclusive ECG findings may lead to a delay in the diagnosis of MI, which is sometimes fatal.^9^ Therefore, we recommend that in cases of BCT with chest pain, the patient should be suspected for MI and monitored with ECG, echocardiography, and cardiac biomarkers at prescribed intervals by the clinicians. Using the highly sensitive cardiac troponin test (hs-TnI assay) is strongly recommended as it reduces the time to diagnose MI from 6 hours to 3 hours.^10^

Motivated by our findings, we searched the literature for similar case reports and found only a few such cases. Christensen et al.^2^ in their 2006 review article, cited 77 cases of MI induced by BCT reported in the literature from 1974 onwards. Among these cases, road traffic accident (RTA) was the most common trauma causing MI, followed by sports injuries; and only 3 cases were due to trauma sustained in a fight. Regarding the age distribution, 82% of the patients were younger than 45 years. The LAD artery was the most common affected vessel, followed by the right coronary artery (RCA).

Also, we found 17 recently reported cases (from the year 2000 to 2018) that showed a similar kind of pattern observed by Christensen et al.^2^ (Table 1). Among these 17 cases, the most common trauma involved was RTA, followed by sports injury. Again, the most common vessel involved was the LAD artery, followed by the RCA, and the majority of the patients (82.4%) were younger than 45 years old. Approximately 59% of the patients did not have any previous history of CAD and its risk factors. Most of the patients (70.6%) had complained of angina on admission.

**Table 1:** Summary of AMI cases induced by BCT

**Case reports**	**Age/sex**	**Mode of chest injury**	**Angina**	**history/risk factors of CAD**	**Outcome**	**Coronary artery & pathology**
Moore^11^	30/M	Sports injury	+	Nil	Survived	LAD—Intimal dissection with thrombus
Vasudevan et al.^12^	21/M	Sports injury	+	IDDM 8 years	Survived	LAD—Dissection
Lai et al.^13^	36/M	BCT	+	Nil	Survived	LAD and LCX—Dissection with thrombus
Janella et al.^9^	31/M	Sports injury	+	Nil	Survived	LAD—Thrombus
Janella et al.^9^	44/M	RTA	+	Nil	Survived	LAD—Aneurysm
Sharma^14^	25/M	RTA	-	Nil	Brought dead	LAD—85% block
Sharma^14^	55/M	Assault	-	Nil	Brought dead	LAD—95% block
Kul et al.^15^	34/M	Assault	+	cigarette smoker	Survived	LAD and LCX—Dissection with thrombus
Lolay and Abdel-Latif^7^	32/M	Sports injury	+	Nil	Survived	RCA—Dissection with thrombus
Abdolrahimi et al.^16^	37/M	RTA	+	Nil	Survived	Proximal part of LAD and RCA dissection
Mubang et al.^17^	25/M	RTA	-	drug abuse	Survived	RCA—Dissection
Sinha et al.^18^	25/F	Kicked by horse	+	Nil	Survived	LAD—Thrombus
Ngam et al.^19^	28/M	RTA	-	N/A	Survived	Proximal RCA dissection
Guo et al.^20^	20/F	Sports injury	+	KD + CA	Survived	LAD—Aneurysms with thrombus
Allemeersch et al.^21^	41/M	RTA	-	N/A	Died	LAD—Thrombus
Fujiwara et al.^22^	60/M	Fall	+	Nil	Died	LAD—Thrombus
Elsayed^23^	50/M	chest hit by strong blow	+	Heavy smoker	Survived	RCA—90% atherosclerotic occlusion

AMI = acute myocardial infarction; BCT = blunt chest trauma; CA = coronary aneurysm; F = female; IDDM = insulin-dependent diabetes mellitus; KD = Kawasaki disease; LAD = left anterior descending; LCX = left circumflex artery; M = male; N/A = not available; RCA = right coronary artery; RTA = roadtraffic accident.

In the present case, the deceased presented with the complaint of chest pain typical of angina without any past history of CAD. The chest trauma was caused by a fight, and the pathology was in the LAD artery. The mechanisms of MI in BCT reported in the literature are coronary artery intimal tearing, thrombosis, coronary atherosclerotic plaque rupture, coronary artery aneurysm and dissection.^9^ In our case, the MI was due to the rupture of atherosclerotic plaque with thrombus formation and occlusion of LAD. Therefore, our findings strongly suggest that BCT may induce MI in the setting of pre-existing coronary artery disease. In one of our previous studies to estimate the prevalence of atherosclerotic coronary stenosis in an asymptomatic population by post-mortem coronary angiography, we found that a significant number of people (29.7%) had coronary artery stenosis.^24^ Also, as previously discussed, the majority of the cases reported in the literature did not have any previous history of coronary artery disease and its risk factors. Hence, we strongly suggest that in every BCT case, the patient should be evaluated for MI irrespective of their risk factors and past history of CAD.

From the forensic perspective, a forensic pathologist needs to find whether the alleged trauma is a direct cause of MI or not. This observation plays a vital role in determining the severity of the punishment that the accused is likely to receive. It is therefore of paramount importance for the forensic pathologists to be aware of such rare presentations. This is one of the reasons that we report this case with a literature review. In this case, the victim was young and had no history of past heart disease, and he suffered from angina immediately after the chest trauma. At the autopsy, 80% occlusion of the proximal portion of the left anterior descending artery (LAD) and the myocardial pallor of the left ventricular anterior wall, interventricular septum, and the apex was observed. The post-mortem histopathological examination clarified that the death was due to AMI consequent to an atherosclerotic plaque rupture with thrombus formation in the LAD. The infiltration of polymorphonuclear neutrophils was also consistent with the chronological history of the trauma. These facts helped us to conclude that chest trauma was the real direct cause of lethal acute myocardial infarction.

## CONCLUSION

It is evident from many studies that even young adults are suffering from CAD without showing any symptoms. Hence, such an asymptomatic adult population is always at risk of MI when they sustain BCT. Thus, in a scenario where a person presents with chest pain after blunt trauma to the chest, he/she should be properly evaluated to rule out MI. Suspicion and early diagnosis may prevent MI-related fatalities. In cases where a person dies of MI due to BCT, the forensic pathologist should be cautious in framing his/her opinion regarding the cause of death and should clarify whether or not the MI is related to the injury sustained in the alleged assault.
